# Emerging patterns in nanoparticle-based therapeutic approaches for rheumatoid arthritis: A comprehensive bibliometric and visual analysis spanning two decades

**DOI:** 10.1515/biol-2025-1071

**Published:** 2025-03-21

**Authors:** Shenwei Xie, Pan Liao, Shuang Mi, Liang Song, Xiaoyuan Chen

**Affiliations:** Department of Rheumatology and Immunology, Hunan University of Medicine General Hospital, HuaiHua, 418000, China; Department of Respiratory and Critical Care Medicine, Shenzhen Yantian District People’s Hospital, Shenzhen, 518000, China

**Keywords:** nanoparticles, rheumatoid arthritis, bibliometrics, visualization study, global trends

## Abstract

The aim of this study is to analyze scientific literature to investigate the current research status, focus areas, and developmental trends in nanoparticle systems for rheumatoid arthritis (RA) therapy. To do that, Research articles published from 2003 to 2023 were retrieved from the Web of Science database, and analysis included quantitative output, distribution by country/region, collaborative publishing data, influential authors, high-yield institutions, keywords, hotspots, and development trends. Visual knowledge maps were generated using VOSviewer and Citespace. Findings reveal a steady increase in publications related to nanoparticle systems for RA therapy, indicating growing global interest. China leads with 487 papers (37.433%), followed by the United States (233, 17.909%), India (179, 13.759%), South Korea (89, 6.841%), and Egypt (50, 3.843%). Active collaboration is observed, particularly between the United States and countries such as China, Germany, Saudi Arabia, India, England, and Pakistan. The Chinese Academy of Sciences ranks first in total articles published (55), with Liu Y from China being the most prolific author. The Journal of Controlled Release emerges as a primary outlet in this field. Primary keyword clusters include “Drug delivery systems,” “Gold nanoparticles,” “Transdermal delivery,” “Angiogenesis,” “Collagen-induced arthritis,” “Rheumatoid arthritis,” “Oxidant stress,” “Dendritic cells,” and “pH sensitive.” Research hotspots with great development potential include “Immunopathological Mechanisms,” “Novel drugs,” and “Smart delivery system.” In conclusion, research on nanoparticle systems for RA therapy has significantly expanded over the past two decades, with a focus on elucidating pathogenetic mechanisms and advancing novel drug delivery strategies anticipated to be prominent in the foreseeable future.

## 1 Introduction

Rheumatoid arthritis (RA) is a chronic systemic autoimmune disease characterized by chronic synovitis, which leads to joint inflammation, synovial hyperplasia, pannus formation, and the destruction of bones and cartilage [1,2]. It affects approximately 1% of the world’s population, with a higher prevalence among females. The epidemiological data on RA based on geographic region and ethnicity is limited, though the female-to-male ratio is around 2:1 [3–7], and the estimated annual incidence is 12 per 100,000 patient-years in East Asia [8]. RA’s prevalence and disease burden vary across geographic regions, generally higher in industrialized countries and urban settings. Common clinical manifestations include persistent arthritis pain, swelling, stiffness, and potential complications such as cardiovascular, pulmonary, psychological, and bone diseases [9,10]. RA is a persistent, progressive, systemic pathology, often associated with autoantibodies like rheumatoid factor and anti-citrullinated protein antibodies [11]. The pathogenesis of RA is believed to involve fibroblasts and cytokines, leading to chronic inflammation, bone erosion, and tissue destruction in the synovial tissue, resulting in various clinical symptoms and injuries [12,13]. Moreover, systemic inflammation can affect multiple organ systems, including the heart, vascular system, kidneys, lungs, and nervous system.

The current therapies for RA are categorized into four mainstream management options: non-steroidal anti-inflammatory drugs (NSAIDs), glucocorticoids, nonbiological disease-modifying anti-rheumatic drugs (DMARDs), and biological DMARDs [14]. However, each drug grouping faces significant challenges, including limited bioavailability, high clearance, and varying degrees of toxicity to normal tissues. While medications like NSAIDs and corticosteroids effectively relieve stiffness and pain, they do not slow disease progression. Over the past 20 years, DMARDs have garnered attention for their effectiveness in reducing disease activity and joint deformity [15]. These include traditional synthetic drugs, biological DMARDs, and novel small molecules. Established DMARDs such as gold onofin, minocycline, azathioprine, and cyclosporine are rarely used in modern therapies. Recent years have seen the emergence of several biological DMARDs, including TNF inhibitors, anti-CD20 antibodies, IL-6 receptor antibodies, RANKL antibodies, and JAK inhibitors [16]. Despite the growing number of treatment options, achieving complete long-term disease remission remains challenging for many patients. While most patients can achieve clinical remission after treatment, some develop refractory RA, requiring lifelong medication. Moreover, RA treatments not only delay the disease but also cause significant adverse effects that can be financially and physically burdensome for patients. Given RA’s chronic nature and its need for new therapeutic approaches, there is a pressing need to explore innovative treatments.

In addition to conventional treatments, structured nanoparticle delivery systems can enhance therapeutic efficacy by prolonging the residence time of the drug [17,18]. Nanotechnology offers advanced carrier systems, notably impacting the delivery of various therapeutic agents, including small molecules, RNA, genes, peptides, and diagnostic imaging agents, thereby improving drug stability and pharmacokinetics [19,20]. Hybridized nanoparticles represent the latest generation of delivery systems, integrating lipid-based and polymeric nanocarriers to combine benefits such as incorporating hydrophilic and hydrophobic drugs, improving stability, and enhancing efficacy [21–23]. In a study published in Nature Nanotechnology, Fang and Zhang reported a multifunctional biosynthesized hybridized nanoparticle formulation with promising potential for treating RA [24]. This formulation, known as Ce-MSCNV, consists of small cerium nanoparticles attached to mesenchymal stem cell nanovesicles [24,25]. Cerium nanoparticles exhibit antioxidant properties, scavenging excess reactive oxygen species (ROS) associated with pathologic inflammation [26,27]. The formulation effectively reduces RA-induced ROS production and modulates macrophage polarization, providing immediate relief from inflammation. Stem cell vesicles deliver immunomodulatory cytokines, promoting immune tolerance and durable disease resolution [28,29]. The combined action of cerium nanoparticles and stem cell vesicles creates a synergistic effect, rapidly treating damaged joints, modulating immune responses, and rebalancing the TH17/Treg cell ratio [30–32]. Overall, as a nano-hybrid therapeutic system with detailed immunomodulation, Ce-MSCNV offers an effective multifactorial approach to treating RA compared to monotherapy.

Chen et al. synthesized macrophage mimetic nanoparticles (M@P-siRNAsT/I) and utilized macrophage mimetic vesicles (MMV) as a delivery vehicle for polymeric biodegradable nanoparticles (PBNPs) and small interfering RNAs targeting tumor necrosis factor (siRNAsT/I) to investigate their potential in treating RA under imaging observation. In their study, they discovered that PBNPs possess catalase functionality, facilitating the decomposition of hydrogen peroxide while simultaneously generating oxygen [33]. The specific gene silencing ability of siRNAsT/I enables M@P-siRNAsT/I to significantly inhibit the expression of pro-inflammatory factors TNF-α and IL-6 in a statistically significant manner [34]. Incorporation of PBNPs and siRNAsT/I into MMV improved its stability and biocompatibility. Both photoacoustic (PA) imaging and fluorescence imaging demonstrated effective targeting of RA by M@P-siRNAsT/I, both *in vitro* and *in vivo*. Furthermore, label-free multispectral PA imaging, micro-CT, and histological analysis indicated that M@P-siRNAsT/I induced statistically significant therapeutic effects for RA, including joint erosion improvement, hypoxia inhibition, and anti-inflammatory effects. Additionally, M@P-siRNAsT/I exhibited a favorable biosafety profile in both *in vitro* and *in vivo* evaluations. These findings suggest that M@P-siRNAsT/I may have potential in PA image-guided therapy for RA.

Despite the considerable research conducted on therapeutic aspects of RA, there remains a noticeable absence of comprehensive and meaningful analyses regarding published trends in the field. Therefore, it is crucial to integrate the current focus and frontiers in nanoparticle systems for RA therapy before pursuing additional basic and clinical research. Bibliometrics employs quantitative analysis using mathematical and statistical methods to review published research findings, offering researchers objective scientific indicators to monitor quantitative changes, distributions, and patterns in existing literature [35,36]. Currently, the extent and depth of nanoparticle research in RA therapy are largely unexplored. To bridge this gap, a dedicated study is underway to compile the current status of joint distraction in RA therapy research, predict prospective keywords and frontiers, and assist researchers in identifying prevailing research trends and frontiers in this emerging field.

## 2 Materials and methods

### 2.1 Data acquisition and search strategies

**Figure 1:**
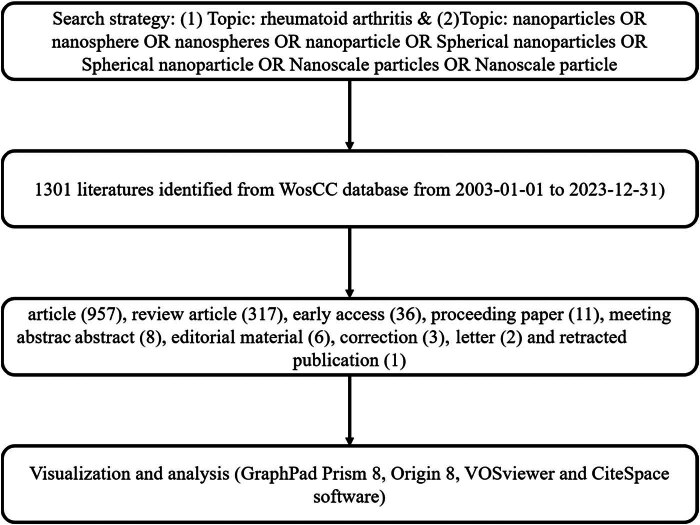
Flowchart depicting the literature selection process.

Publications pertaining to nanoparticle research in RA treatment were retrieved using the SCI Extension database within the Web of Science Core Collection by Clarivate Analytics. Subsequently, studies related to nanoparticle systems for RA therapy were identified, and bibliometric and visualization analyses were conducted following established methodologies from previous studies. The search parameters were set from January 1, 2003, to December 31, 2023, with the search formula structured as follows: TS = (rheumatoid arthritis) AND TS = (nanoparticles OR nanospheres OR nanospheres OR nanoparticles OR Spherical nanoparticles OR Spherical nanoparticle OR Nanoscale particles OR Nanoscale particle). Additionally, the publication criteria included: [1] publications primarily focused on nanoparticles in RA therapeutics, [2] literature types limited to articles and reviews, and [3] papers written in English. The exclusion criteria included: [1] publications that were not related to immunology research in the context of RA; [2] articles classified as meeting abstracts, conference proceedings, corrections, book chapters, letters, news articles, or similar non-peer-reviewed content (Figure 1). For the included studies, we extracted comprehensive data, including the publication year, title, authorship, nationalities, affiliations, abstract, keywords, and journal names. This information was saved in .txt format for subsequent analysis. Data extraction was carried out independently by coauthors Xie and Liao. Any disagreements were resolved through consultation with experts to reach a consensus. The final dataset was imported into CiteSpace and VOSviewer for visualization and bibliometric analysis, ensuring a robust and transparent approach toward data handling and analysis in accordance with PRISMA guidelines.

The authors extracted basic information regarding the publications, including details such as journals, titles, authors, keywords, institutions, countries/regions, publication dates, as well as comprehensive statistics such as total citations, H-index, and average citation counts. This information was then imported into Excel 2021. Subsequently, bibliometric analyses and visualizations were conducted using a suite of software applications, including GraphPad Prism 8, Origin 2021, and VOSviewer (version 1.6.14, Leiden University, Leiden, The Netherlands) [37], and CiteSpace (version 6.2.4) [38]. These tools played a pivotal role in dissecting and visualizing the intricate landscape of publications related to nanoparticle systems for RA therapy, offering a comprehensive perspective on the scholarly contributions in this domain.

### 2.2 Bibliometric analysis and visualization

Bibliometrics entails studying interconnected bodies of literature. It involves analyzing and visualizing links between research topics, researchers, affiliations, or journals. Initially, annual publication trends and relative research interest (RRI) were graphically represented using the curve-fitting function in GraphPad Prism 8. RRI is calculated as the number of papers in a specific field divided by the total number of papers in all fields in a given year, offering insights into the prominence of the field relative to others. For the world map analysis, a methodology based on previous research was utilized [39]. Furthermore, the total number of publications for the top ten countries between 2003 and 2023, along with global trend projections, were analyzed using Origin 2021 software.

An in-depth examination of pertinent studies was undertaken utilizing VOSviewer and Citespace software tools to elucidate collaborations (co-authors), themes (terminology co-occurrence), and citation patterns (bibliographic coupling). This entailed scrutinizing country/region and institution collaborations, overlaying journal biplots, analyzing author collaborations, investigating co-cited authors, and performing cluster analyses. Furthermore, co-cited references and keywords were meticulously identified and assessed to highlight those with significantly higher citations. By configuring relevant parameters as described above, the analysis ensured robust and precise exploration of a substantial volume of literature data pertaining to nanoparticle systems for RA therapy.

Moreover, this investigation utilized VOSviewer to construct and visualize the bibliometric network, facilitating the acquisition of more comprehensive insights, including (1) co-citation analysis of journals and references, and (2) keyword co-occurrence analysis. In the graphical representation produced by VOSviewer, each node corresponds to an entity containing co-cited references and keywords. The node’s size corresponds to the number of publications associated with it, while its color denotes the corresponding publication year. The thickness of the connecting lines between nodes indicates the strength of collaborative or co-citation relationships, offering an intuitively visual portrayal of the intricate interconnections within bibliographic data. This approach enhances the depth of understanding regarding thematic and conceptual associations within the realm of nanoparticle systems for RA therapy.

## 3 Results

### 3.1 Global contribution to the field

**Figure 2:**
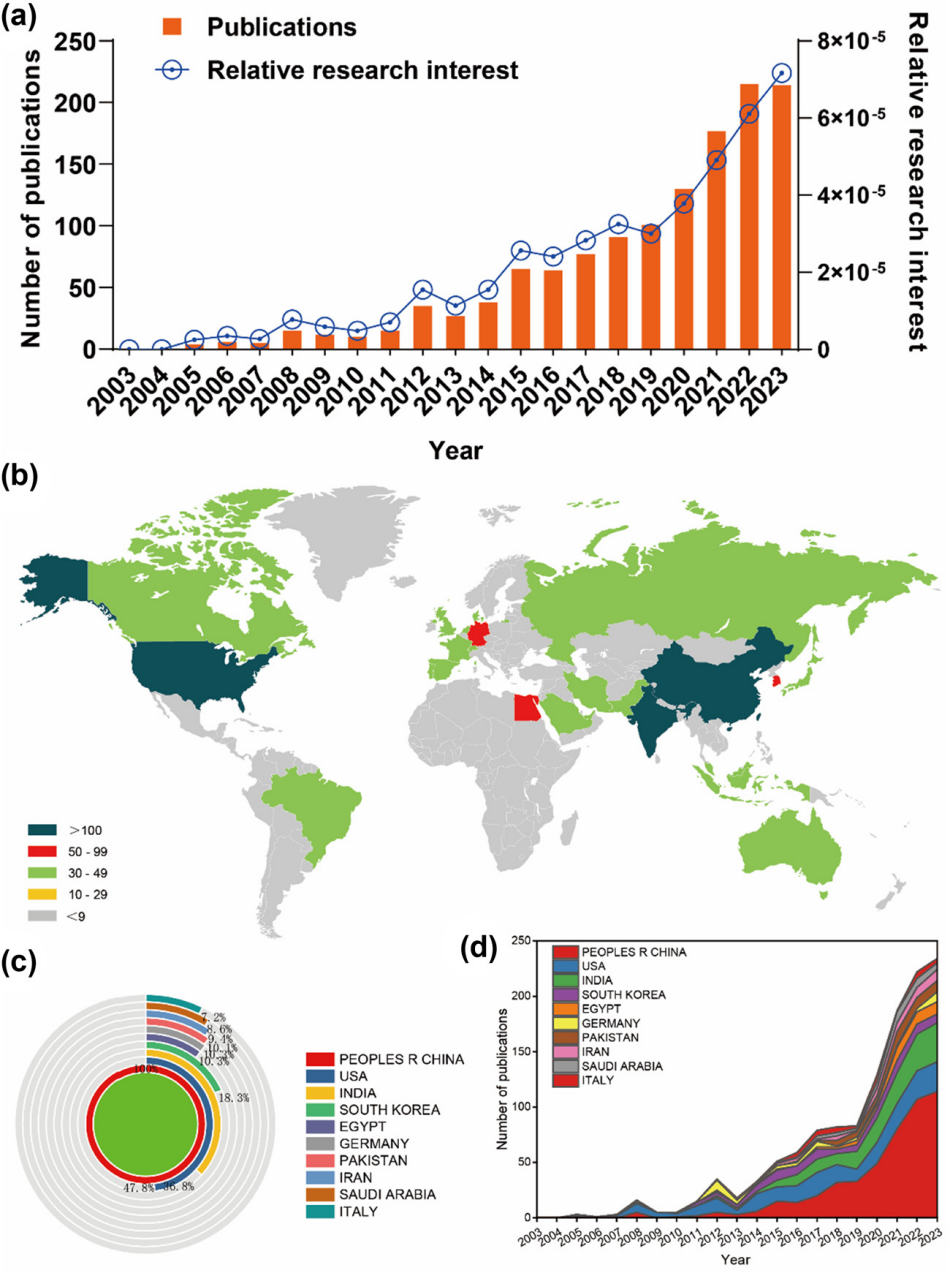
Global trends and the geographical landscape of research on nanoparticles in RA. (a) Annual publication statistics concerning nanoparticles in RA research. (b) A global map illustrating the dispersion of such research. The cumulative (c) and yearly (d) publication counts across the top ten most prolific countries between 2013 and 2023.

Based on a meticulously crafted publication search strategy (outlined in Figure 1), a total of 1,301 publications met the established criteria and were included in the final analysis. The number of publications per year from 2003 to 2023 exhibited a gradual and fluctuating trend, surging from a modest 4 articles to an impressive 215+ articles (as depicted in Figure 2a). Concurrently, the RRI demonstrated a relatively stable trend around the baseline level over the same period (as illustrated in Figure 2a). Overall, contributions in the field of RA therapeutic research involving nanoparticles emanated from 73 different countries/regions. Remarkably, China led the way with the largest share of 487 publications, constituting 37.43% of the total. This was followed by the USA (233 publications, 17.91%), India (179 publications, 13.76%), and South Korea (89 publications, 6.84%) (depicted in Figure 2b and c). Furthermore, Figure 2d underscores the evolving landscape of publication contributions, with China taking the lead from 2015 to 2023 by surpassing the United States, continually achieving new highs and forging ahead. However, in recent years, other countries have also made significant strides and remain competitive in advancing research endeavors.

### 3.2 Distribution of countries/regions and institutions

**Figure 3:**
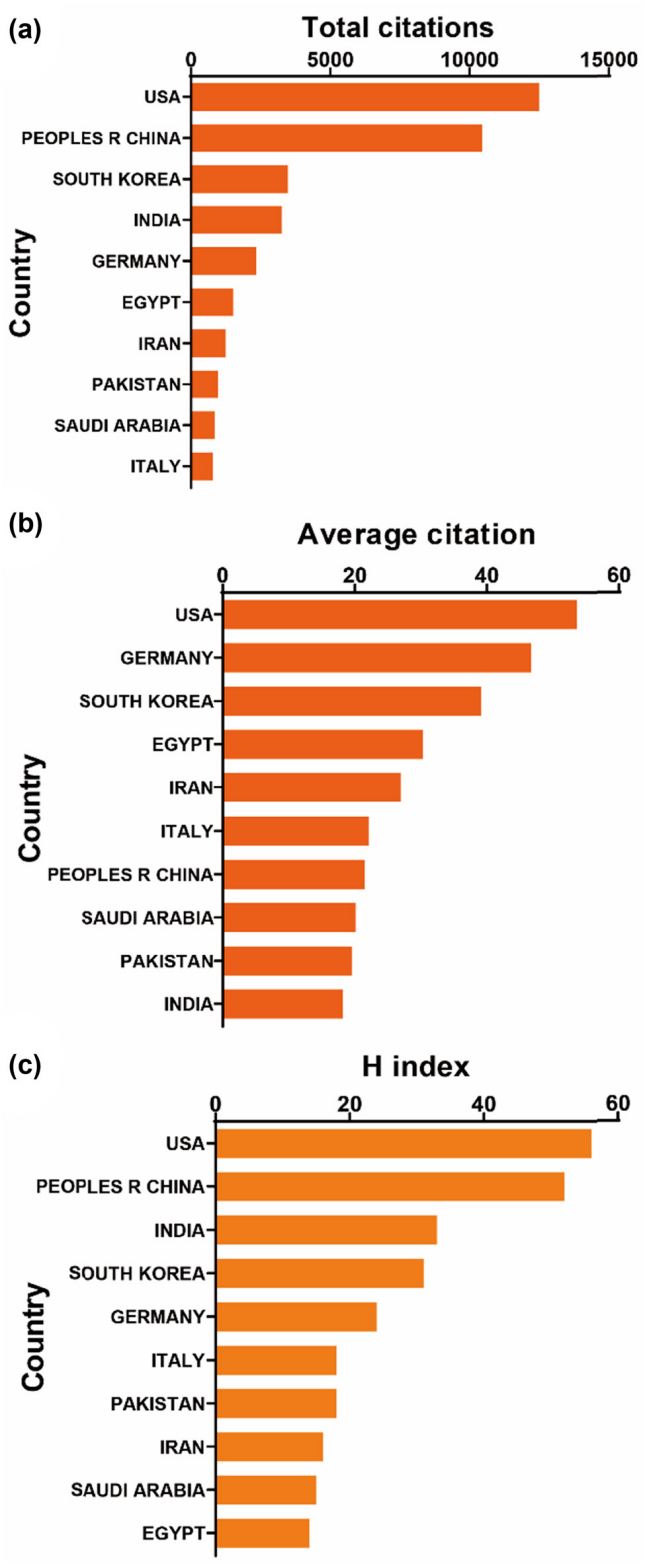
(a) The top ten countries/regions with the highest total citations in research on nanoparticles for RA. (b) The top ten countries/regions with the highest average citations per publication in research on nanoparticles for RA. (c) The top ten countries/regions with the highest publication H-index in research on nanoparticles for RA.

**Figure 4:**
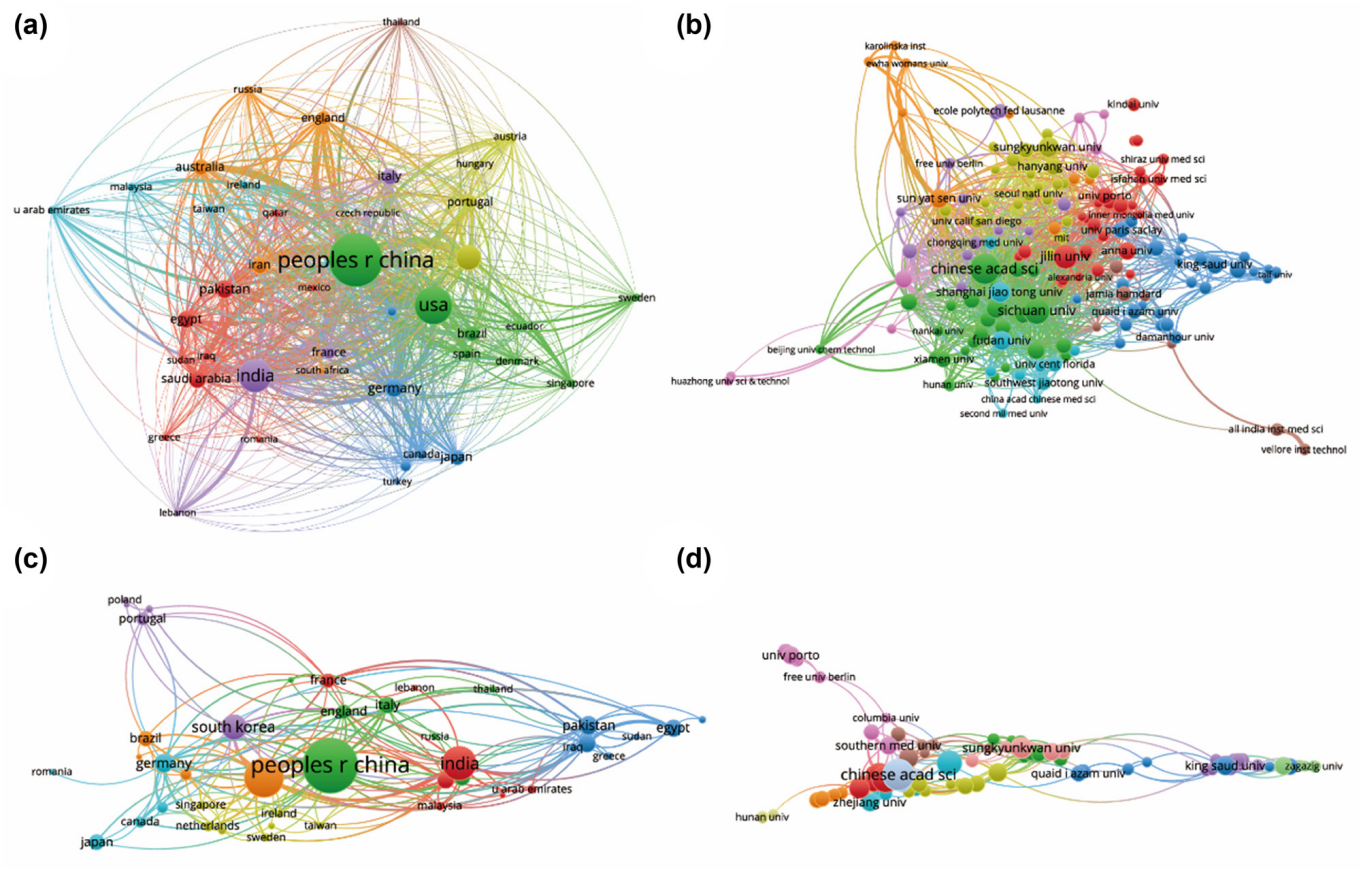
Mapping the involvement of countries, regions, and institutions in research on nanoparticles for RA. (a) Examination of country and regional collaborations utilizing VOSviewer. (b) Assessment of institutional collaborations using VOSviewer. (c) Analysis of authorship-country collaborations via VOSviewer. (d) Evaluation of authorship-institution collaborations via VOSviewer. Node sizes represent countries/regions, scaled by their publication counts. Connecting lines indicate collaboration, with thickness reflecting the strength of cooperation; thicker lines denote closer collaboration.

All 1,301 publications originated from a diverse array of 73 countries and engaged 1,760 distinct organizations. Notably, the top ten countries/regions exhibit extensive global distribution, primarily spanning North America, Asia, and Western Europe (Figure 2b). China emerges as a particularly formidable contributor, representing over one-third of the total publications, markedly surpassing other nations. Furthermore, Figure 3 illustrates that the USA garners the highest total number of citations (12,496) and boasts a notable H index of 56, surpassing all other countries. With the highest average citation rate (53.63), the USA, along with Germany and South Korea, solidifies their pivotal positions in publications. Together, these three countries account for more than 27% of the total publications, underscoring their considerable influence in the field. Subsequently, the collaborative patterns between countries/regions are depicted in Figure 4a, with China demonstrating robust collaboration, as evidenced by the size of its node.

As demonstrated in Table 1, the top ten most productive institutions are prominently situated in China, Egypt, France, America, and South Korea. It is noteworthy that despite not leading in total publications, Harvard University boasts a significantly higher average citation rate compared to other institutions. Moreover, the analysis of institutional collaborations, as depicted in Figure 4b, highlights Sichuan University, Shanghai Jiao Tong University, and the Chinese Academy of Sciences as leaders in collaborations with other research entities.

**Table 1:** Top ten institutions published literature related to nanoparticles in RA research

Rank	Institution	Article counts	Percentage (%)	Country
1	Chinese Academy of Sciences	55	4.228	China
2	Egyptian Knowledge Bank (EKB)	50	3.843	Egypt
3	Sichuan University	36	2.767	China
4	Jilin University	27	2.075	China
5	Centre National De La Recherche Scientifique	23	1.768	France
6	Shanghai Jiao Tong University	23	1.768	China
7	Harvard University	21	1.614	
8	Southern Medical University China	20	1.537	China
9	Fudan University	19	1.46	China
10	Sungkyunkwan University	19	1.46	South Korea

Table 2 delineates the top ten funders in terms of the number of articles published in the field of nanoparticles in RA studies, with the National Natural Science Foundation of China (NSFC) clinching the top spot with a substantial number of 313 articles, accounting for 24.06%. This is followed by the National Institutes of Health (USA) and the United States Department of Health and Human Services, both with 94 articles (7.23%). Subsequent to these, the China Postdoctoral Science Foundation follows closely with 42 articles (3.23%). Support from these relevant organizations has significantly contributed to the advancement of research in this area.

**Table 2:** Top ten funds related to nanoparticles in RA research

Rank	Journal	Article counts	Percentage (%)
1	National Natural Science Foundation of China	313	24.058
2	National Institutes of Health (USA)	94	7.225
3	United States Department of Health Human Services	94	7.225
4	China Postdoctoral Science Foundation	42	3.228
5	European Union	39	2.998
6	National Research Foundation of Korea	34	2.613
7	National Natural Science Foundation of Guangdong Province	25	1.922
8	Fundamental Research Funds for The Central Universities	24	1.845
7	National Science Foundation	23	1.768
10	Fundacao Para A Ciencia E a Tecnologia Fct	20	1.537

### 3.3 Analysis of journals and research areas

**Figure 5:**
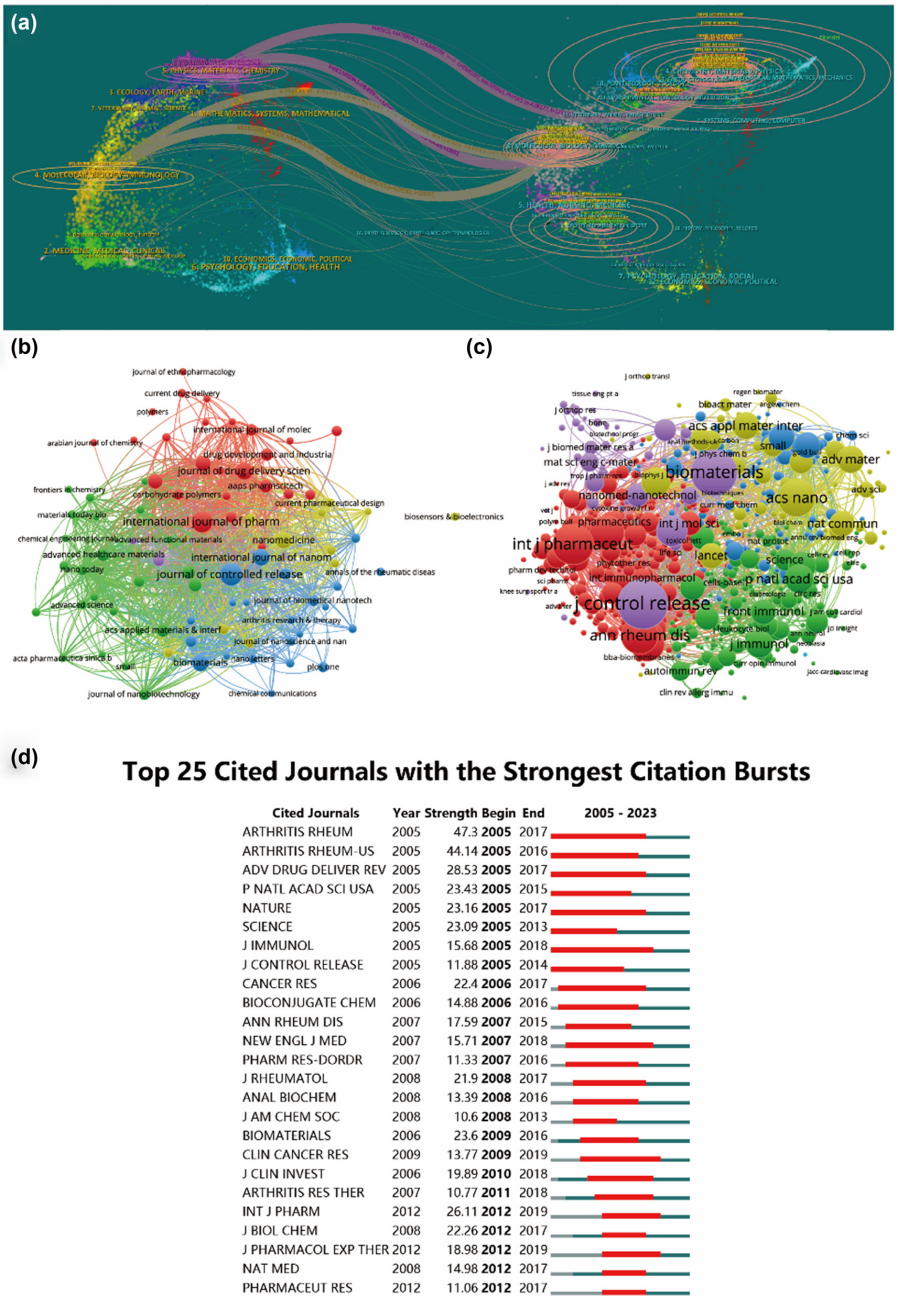
Articles published in various journals on nanoparticles in RA research. (a) Dual-map overlay showcasing journals relevant to tissue engineering and regenerative medicine for rotator cuff injuries. (b) Bibliographic analysis of journals using VOSviewer. (c) Network map illustrating journals that were co-cited, as analyzed by VOSviewer. (d) Presentation of the top 25 cited journals exhibiting the most significant citation bursts in related publications.

Between 2003 and 2023, a total of 1,301 articles were published across 443 journals. Table 3 highlights the top ten journals with the highest number of publications alongside their most recent impact factors (IF). Leading the pack is the Journal of Controlled Release with 47 publications, constituting 3.613% of all articles, followed by the International Journal of Pharmaceutics (41 publications, 3.151%), International Journal of Nanomedicine (30 articles, 2.306%), Pharmaceutics (28 articles, 2.152%), and Biomaterials (27 articles, 2.075%). Remarkably, among these top ten journals, ACS Nano boasted the highest IF of 17.1, trailed closely by Biomaterials (14.0) and Journal of Controlled Release (10.8). The distribution of articles across various journals focusing on nanoparticles in RA research is depicted in Figure 5a–d.

**Table 3:** Top ten most productive journals related to nanoparticles in RA research

Rank	Journal	Articles count	Percentage (%)	IF (2023)
1	Journal of Controlled Release	47	3.613	10.8
2	International Journal of Pharmaceutics	41	3.151	5.8
3	International Journal of Nanomedicine	30	2.306	8.0
4	Pharmaceutics	28	2.152	5.4
5	Biomaterials	27	2.075	14.0
6	Journal of Drug Delivery Science and Technology	24	1.845	5.0
7	ACS Nano	18	1.384	17.1
8	Journal of Materials Chemistry B	18	1.384	7.0
9	ACS Applied Materials Interfaces	17	1.307	9.5
10	Drug Delivery	17	1.307	6.0

Moreover, the identified publications were categorized into 62 different research areas. Among the top ten most represented research areas, Pharmacology Pharmacy accounted for the majority with 455 records, comprising 34.973% of all articles, followed by Chemistry (350 or 26.902%) and Materials Science (347 or 26.672%) (as illustrated in Table 4). Additionally, Figure 5a features a bi-map overlay of journals to demonstrate those related to tissue engineering and regenerative medicine for rotator cuff injuries.

**Table 4:** Top ten well-represented research areas

Rank	Research areas	Records	Percentage (%)
1	Pharmacology pharmacy	455	34.973
2	Chemistry	350	26.902
3	Materials science	347	26.672
4	Science technology and other topics	303	23.29
5	Engineering	119	9.147
6	Biochemistry and molecular biology	104	7.994
7	Research experimental medicine	93	7.148
8	Physics	81	6.226
9	Biotechnology applied microbiology	70	5.38
10	Immunology	62	4.766

### 3.4 Authors analysis

**Figure 6:**
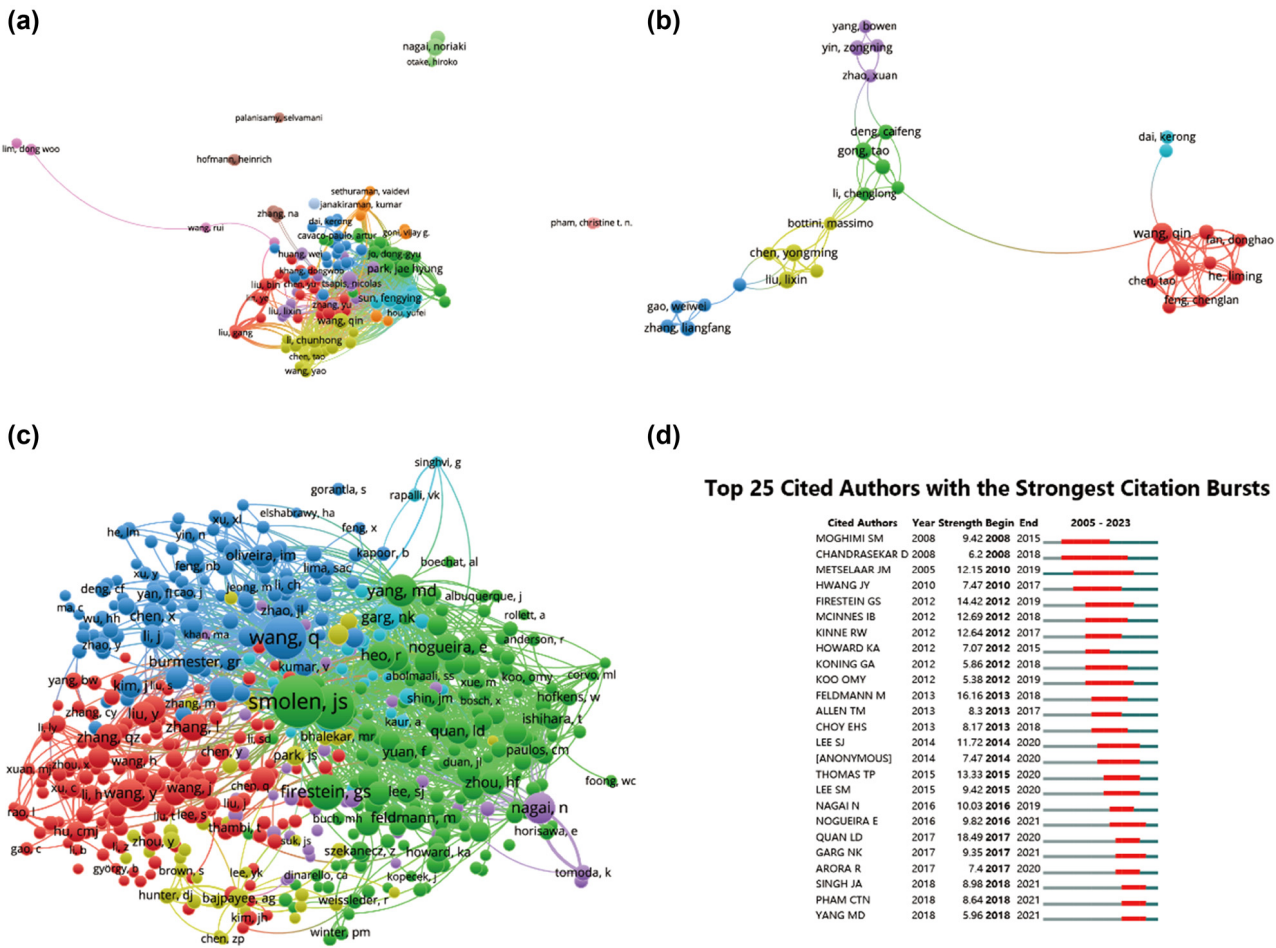
Network visualization of author collaboration in nanoparticle research for RA. (a) Author collaboration analysis conducted using VOSviewer. (b) Network visualization diagram illustrating authorship–author relationships according to VOSviewer analysis. (c) Network visualization diagram depicting co-cited-author relationships based on VOSviewer analysis. (d) Presentation of the top 25 cited authors exhibiting the most significant citation bursts in publications related to tissue engineering and regenerative medicine for rotator cuff injuries. Author collaborations are represented by nodes, with node size proportional to the number of collaborations. Collaboration connections are depicted by lines between nodes.

Table 5 enumerates the top ten authors who have made noteworthy contributions in the realm of nanoparticles in RA research. Foremost among them is Liu Y, with 20 publications, closely trailed by Park JH, Wang Q, and Zhang Y, each with 17 publications. Furthermore, to visually depict collaborative relationships between researchers, author collaborations were analyzed and presented in Figure 6. Additionally, a co-cited author network visualization graph (Figure 6b and c) was generated. In these visualizations, nodes represent authors, with their size corresponding to the number of collaborations, while lines between nodes signify collaboration connections. Figure 6d showcases the top 25 cited authors, all of whom have significantly influenced research in their respective fields.

### 3.5 Citation and co-citation analysis of reference

**Figure 7:**
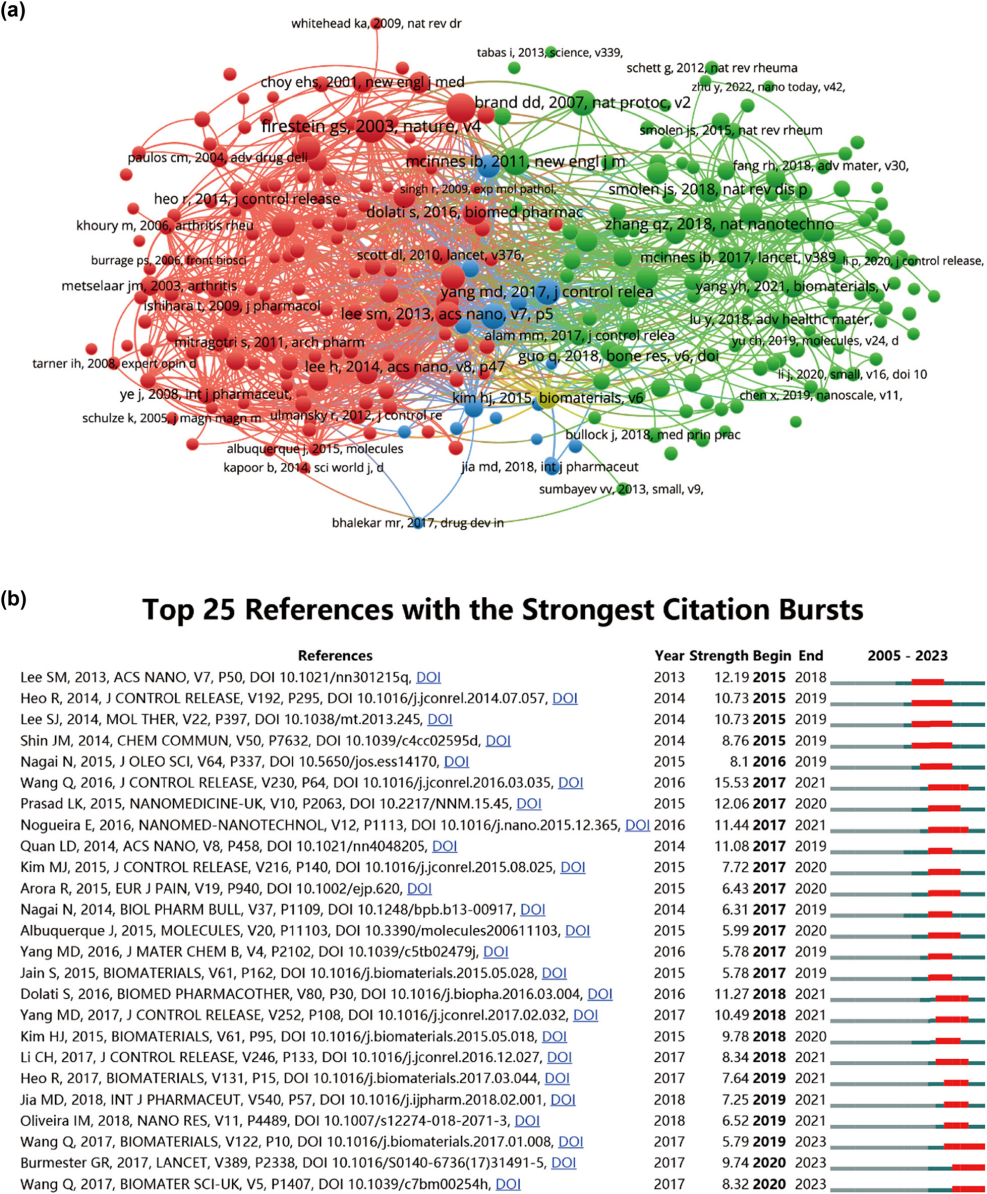
Mapping of references in nanoparticle research for RA. (a) Network map illustrating reference analysis conducted using VOSviewer. (b) Presentation of the top 25 references exhibiting the most significant citation bursts in related publications.

We conducted a thorough analysis of the literature in the field, focusing on papers with over 25 citations, totaling 106 articles, which were then visualized using VOSviewer (as depicted in Figure 7a). Table 6 provides details of the top ten cited research papers. Topping the list is “*In vivo* tumor targeting and spectroscopic detection with surface-enhanced Raman nanoparticle tags,” which amassed the highest number of citations at 1,972. Following closely is “Neutrophil membrane-coated nanoparticles inhibit synovial inflammation and alleviate joint damage in inflammatory arthritis,” securing second place with 516 citations. In third place is “Antiangiogenic properties of gold nanoparticles,” garnering 378 citations. Additionally, Table 7 outlines the top ten cited review articles. Both “Impact of albumin on drug delivery - New applications on the horizon” and “Biological properties of ‘naked’ metal nanoparticles” received the highest number of citations, each accumulating 650. Subsequently, “Clinical impact of serum proteins on drug delivery” secured third place with 292 citations. The fourth most cited paper was “Cell penetrating peptides: a concise review with emphasis on biomedical applications,” with 222 citations. Moreover, references with citation bursts can serve as valuable indicators of frequently cited literature in a specific field over time [40]. In this study, Figure 7b illustrates the top 25 references that demonstrated the most significant citation bursts, along with the corresponding duration of these bursts. Notably, the article “Targeted delivery of low-dose dexamethasone using PCL- PEG micelles for effective treatment of rheumatoid arthritis,” published in 2016, topped the list with an intensity of 15.53.

**Table 7:** Top ten review articles with the most citations in the field of nanoparticles in RA research

Rank	Title	First author	Journal	IF (2023)	Publication year	Total citations
1	Impact of albumin on drug delivery – New applications on the horizon	Elsadek, B	Journal of Controlled Release	10.8	2012	650
2	Biological properties of “naked” metal nanoparticles	Bhattacharya, R	Advanced Drug Delivery Reviews	16.1	2008	650
3	Clinical impact of serum proteins on drug delivery	Kratz, F	Journal of Controlled Release	10.8	2012	292
4	Cell penetrating peptides: A concise review with emphasis on biomedical applications	Derakhshankhah, H	Biomedicine & Pharmacotherapy	7.5	2018	222
5	Delivery of drugs and biomolecules using carbon nanotubes	Vashist, SK	Carbon	10.9	2011	216
6	Biomimetic nanoparticles for inflammation targeting	Jin, K	Acta Pharmaceutica Sinica B	14.5	2018	198
7	Nanomaterials for nanotheranostics: tuning their properties according to disease needs	Wong, XY	ACS Nano	17.1	2020	194
8	Recent advances in design of functional biocompatible hydrogels for bone tissue engineering	Xue, X	Advanced Functional Materials	19.0	2021	179
9	A review of therapeutic challenges and achievements of methotrexate delivery systems for treatment of cancer and RA	Abolmaali, SS	Cancer Chemotherapy and Pharmacology	3.0	2013	174
10	Methotrexate: a detailed review on drug delivery and clinical aspects	Khan, ZA	Expert Opinion on Drug Delivery	6.6	2012	168

### 3.6 Co-occurrence analysis of keywords

**Figure 8:**
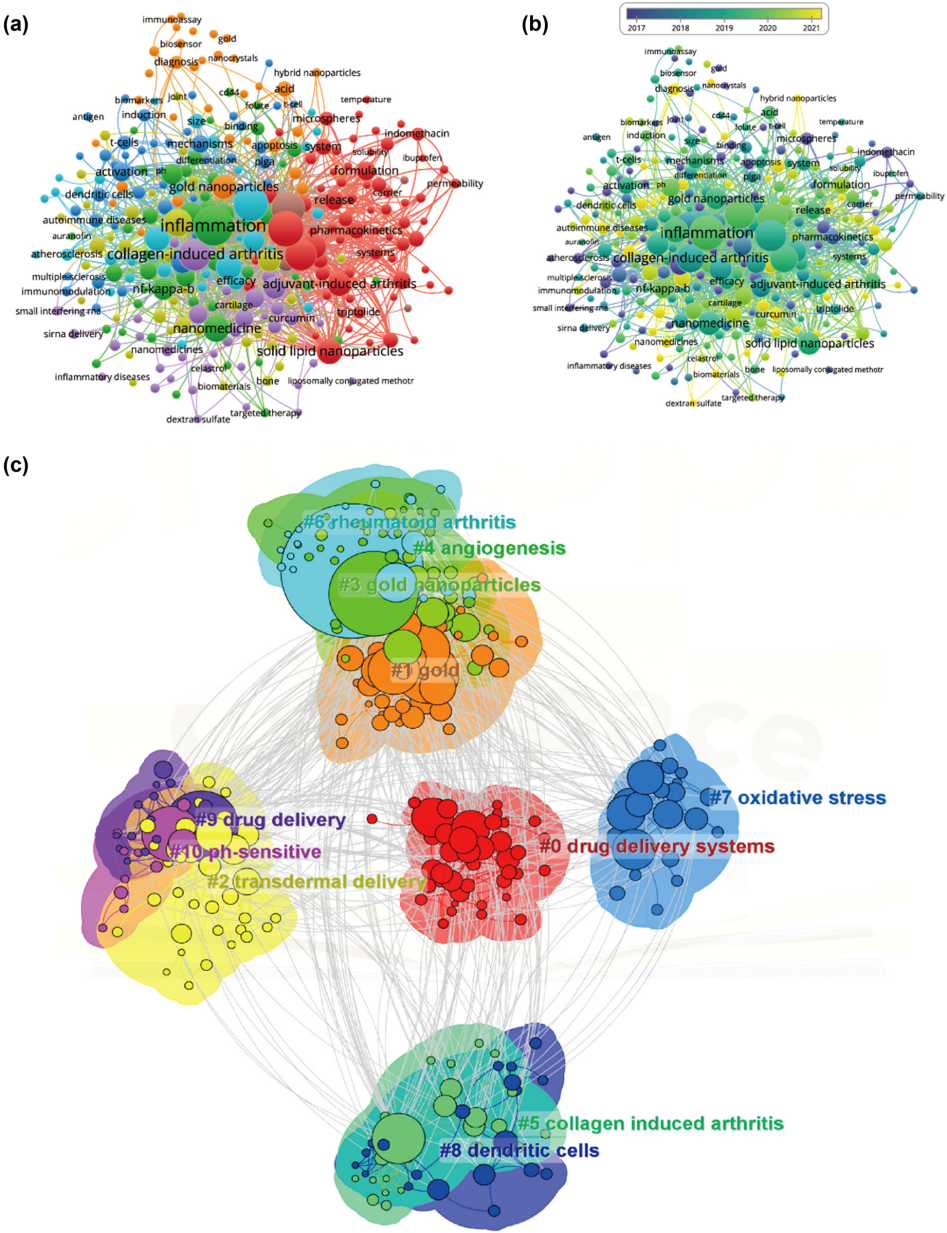
Mapping of keywords in studies concerning nanoparticles in RA research. (a) Network visualization of keywords using VOSviewer, with point size representing frequency. (b) Distribution of keywords based on mean frequency of appearance; keywords in yellow emerged later than those in blue. (c) Visualization of keyword clustering from 2003 to 2023.

In this study, Figure 8 illustrates the co-occurrence cluster analysis of keywords using CiteSpace and VOSviewer to capture the research frontiers in the field. Initially, VOSviewer constructed a network map to analyze the distribution of keywords based on their average year of publication, where dark blue indicates earlier years and yellow indicates later years (as depicted in Figure 8b). A total of 311 keywords were identified, with the five most frequent keywords being: inflammation (total link strength: 1,044), drug-delivery (total link strength: 839), delivery (total link strength: 793), methotrexate (total link strength: 756), and collagen-induced arthritis (total link strength: 714). Most keywords were published before 2020, while terms such as green synthesis and extracellular vesicles emerged relatively recently after 2021. Subsequently, these clusters were divided into 11 different aspects, as shown in Figure 8c, including drug delivery systems, gold, transdermal delivery, gold nanoparticles, angiogenesis, collagen-induced arthritis, RA, oxidative stress, dendritic cells, drug delivery, and pH-sensitive.

**Figure 9:**
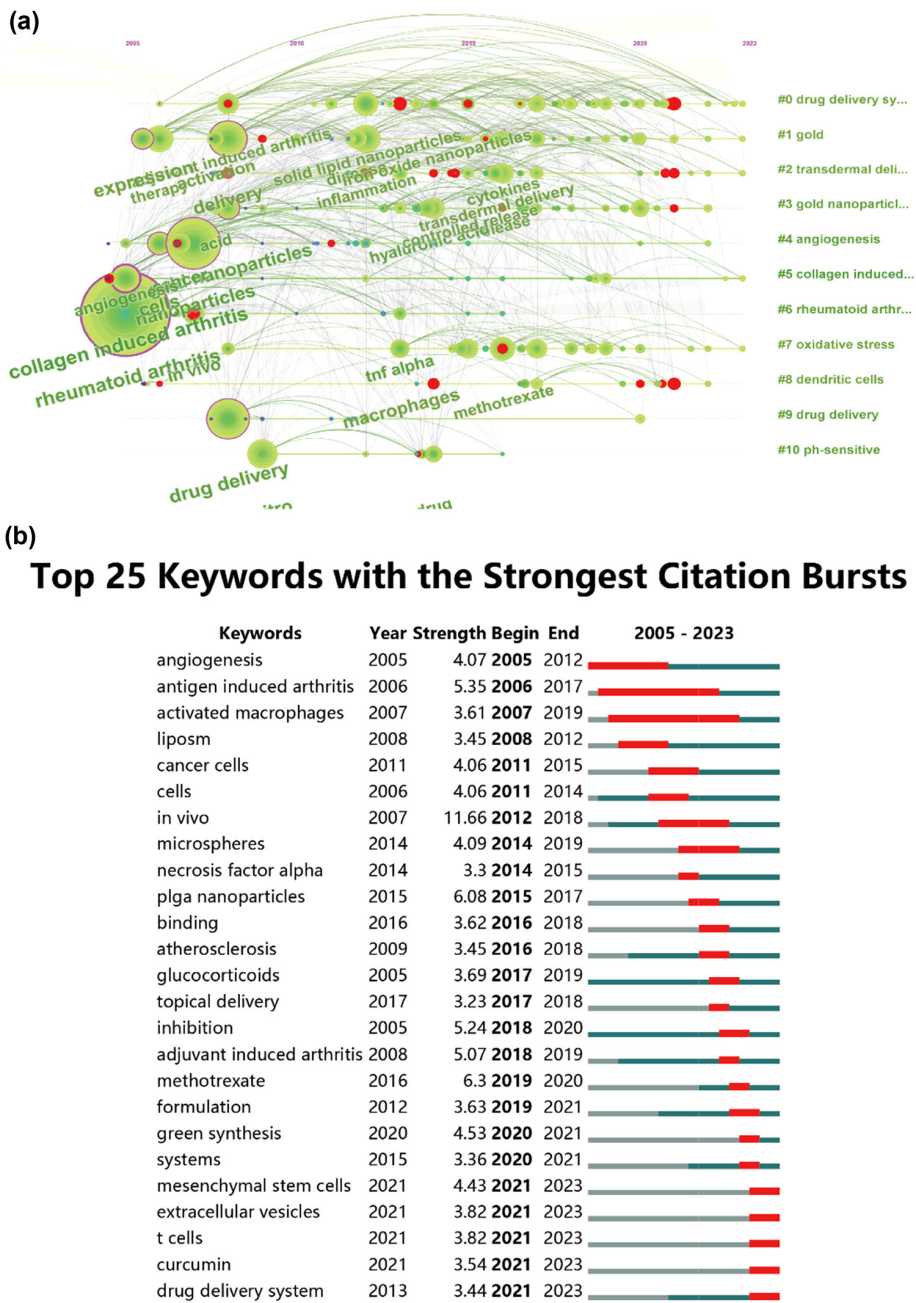
(a) Visualization of keyword timeline from 2013 to 2023. (b) Presentation of the top 25 keywords displaying the most significant citation bursts in publications.

Furthermore, Figure 9a illustrates the temporal dynamic evolution of the keyword clusters as depicted by CiteSpace. In total, 11 clusters were identified, all of which represent current research hotspots. Moreover, CiteSpace’s algorithm was utilized to scrutinize keyword bursts and identify the top 25 keywords with the most pronounced citation bursts (as shown in Figure 9b). Notably, “*in vivo*” exhibited the strongest citation burst with an intensity of 11.66, followed by “poly(lactic-co-glycolic) acid (PLGA) nanoparticles” (intensity = 6.08) and “antigen-induced arthritis” (intensity = 1.78). “Activated macrophages” showed the longest burst duration, spanning 12 years from 2007 to 2019. Additionally, keywords such as “tumor necrosis factor-alpha” (2014–2015), “atherosclerosis” (2016–2018), “glucocorticoids” (2017–2019), “inhibition” (2018–2020), and “formulation” (2019–2021), among others, also exhibited significant burst periods. Interestingly, “mesenchymal stem cells,” “extracellular vesicles,” “curcumin,” “drug delivery system,” etc., are keywords that have recently experienced a surge in citations, indicating that research in these areas may represent future research hotspots.

## 4 Discussion

### 4.1 Trends of nanoparticle system for RA therapy research

Our research spanning from January 1, 2003, to December 31, 2023, reveals a consistent increase in the annual publication count, accompanied by a slight uptick in relative research interest (RRI) in recent years. A diverse pool of approximately 70 countries has contributed to this field of study, with China leading the pack with 487 publications, representing 37.43% of the total output. Notably, Figure 3a underscores the USA’s prominence, leading in total citations, boasting the highest H-index, and exhibiting the highest average citation rate, thus underscoring its pivotal role and exceptional quality contributions to the field. Interestingly, Germany and South Korea trail closely behind in average citations. In the realm of research institutions, the Chinese Academy of Sciences, Egyptian Knowledge Bank (EKB), and Sichuan University emerge as dynamic entities propelling the frontiers of research. It is noteworthy that the top ten institutions predominantly hail from developed nations. Collectively, these findings signal an imminent surge in studies offering profound insights and comprehensive understanding of nanoparticle systems in RA therapeutic research.

### 4.2 Status and quality of authors, journals, and studies publications

In addition to the institutional analysis, our examination extended to the realm of journals, with findings presented in Table 3. Notably, Journal of Controlled Release, International Journal of Pharmaceutics, and International Journal of Nanomedicine emerged as the most prolific publishers in this domain. When considering IF, ACS Nano, Biomaterials, and Journal of Controlled Release garnered the highest IF values, indicating their significance in disseminating high-quality research. It is foreseeable that these top ten journals will continue to serve as primary platforms for cutting-edge research, given their combined quantitative and qualitative prowess. Moreover, a journal-based co-citation analysis was conducted to gauge publication impact and quantify total citations. Figure 5a illustrates that Journal of Controlled Release has made the most substantial contribution to the field. Among the top ten research directions, diverse domains such as clinical, biological, and chemical research were identified, highlighting prevalent interdisciplinary synergies within the field.

Shifting focus to authors, we present the most prolific authors in Table 5. These top-ranked authors have authored numerous studies that are anticipated to drive future research in RA therapeutic applications of nanoparticle systems. Furthermore, collaboration analysis depicted in Figure 6a reveals relatively frequent collaborations among authors within the same country, suggesting the importance of bolstering international academic connections and exchanges. Noteworthy is the emergence of Smolen JS, Wang Q, Mcinnes IB, and Yang MD as highly cited distinguished authors, as shown in Figure 6c. This reflects the international acclaim and recognition garnered by these researchers within the field.

**Table 5:** Top ten authors with the most publications on nanoparticles in RA research

Rank	Highly published authors	Article counts	Percentage (%)
1	Liu Y	20	1.537
2	Park JH	17	1.307
3	Wang Q	17	1.307
4	Zhang Y	17	1.307
5	Zhang N	16	1.23
6	Wang Y	14	1.076
7	Li CH	13	0.999
8	Li J	13	0.999
9	Nagai N	12	0.922
10	Chen X	11	0.846

**Table 6:** Top ten research articles with the most citations in the field of nanoparticles in RA research

Rank	Title	First author	Journal	IF	Publication year	Total citations
1	*In vivo* tumor targeting and spectroscopic detection with surface-enhanced Raman nanoparticle tags	Qian, XM	Nature Biotechnology	46.9	2008	1,972
2	Neutrophil membrane-coated nanoparticles inhibit synovial inflammation and alleviate joint damage in inflammatory arthritis	Zhang, QZ	Nature Nanotechnology	38.3	2018	516
3	Antiangiogenic properties of gold nanoparticles	Mukherjee, P	Clinical Cancer Research	11.5	2005	378
4	Interspecies communication between plant and mouse gut host cells through edible plant derived exosome-like nanoparticles	Mu, JY	Molecular Nutrition & Food Research	5.2	2014	363
5	Gold nanoparticles: A revival in precious metal administration to patients	Thakor, AS	Nano Letters	10.8	2011	358
6	Synergistic Oxygen generation and ROS scavenging by manganese ferrite/ceria co-decorated nanoparticles for RA treatment	Kim, J	Acs Nano	17.1	2019	286
7	Chitosan/siRNA nanoparticle-mediated TNF-α knockdown in peritoneal macrophages for anti-inflammatory treatment in a murine arthritis model	Howard, KA	Molecular Therapy	12.4	2009	236
8	Clearance of pathological antibodies using biomimetic nanoparticles	Copp, JA	Proceedings of the National Academy of Sciences of the United States of America	11.1	2014	206
9	Targeted silver nanoparticles for RA therapy via macrophage apoptosis and Re-polarization	Yang, YH	Biomaterials	14.0	2021	194
10	Targeted chemo-photothermal treatments of RA using gold half-shell multifunctional nanoparticles	Lee, SM	Acs Nano	17.1	2013	194

The impact of published literature was evaluated through citation analysis of documents (depicted in Figure 5a) and same-citation network analysis (illustrated in Figure 5c). Notably, the most cited article originated from NATURE, presenting a comprehensive nanoparticle-based anti-inflammatory strategy for the treatment of RA [41]. The review by Qiang Guo et al. meticulously dissects the etiology and pathology across specific stages: the (i) trigger, (ii) maturation, (iii) target, and (iv) fulminant phases, elucidating synovial hyperplasia, cartilage damage, bone erosions, and systemic consequences. It delves into recent advancements in understanding RA pathogenesis, disease-modifying medications, and provides insights into next-generation RA treatments. Among the top five most cited articles, the majority focus on clinically oriented topics, emphasizing clinical treatments, systematic evaluations, and experimental clinical studies in sports medicine. Additionally, in the co-citation analysis of references, the pivotal publication authored by Smolen et al. warrants considerable attention.

### 4.3 Strengths and limitations

While this study offers valuable insights and direction regarding nanoparticle systems in RA therapy, it is essential to recognize its limitations. First, delving into intricate details of nanoparticle design and application, including particle characteristics, treatment duration, drug release efficiency, nanoparticle degradation mechanisms, and their impact on RA treatment outcomes, presents challenges. Second, the selection of publications may be biased due to constraints of chosen databases and languages. For instance, publications from reputable sources like Cochrane, Embase, and non-English journals may have been overlooked. Finally, the latest high-quality papers may not have garnered sufficient citations yet, potentially creating a gap between bibliometric analyses and real-world advancements. Therefore, researchers are encouraged to stay vigilant for the latest publications, particularly those in non-English languages, to ensure a comprehensive and current understanding of the field.

## 5 Research hotspots and frontiers

**Figure 10:**
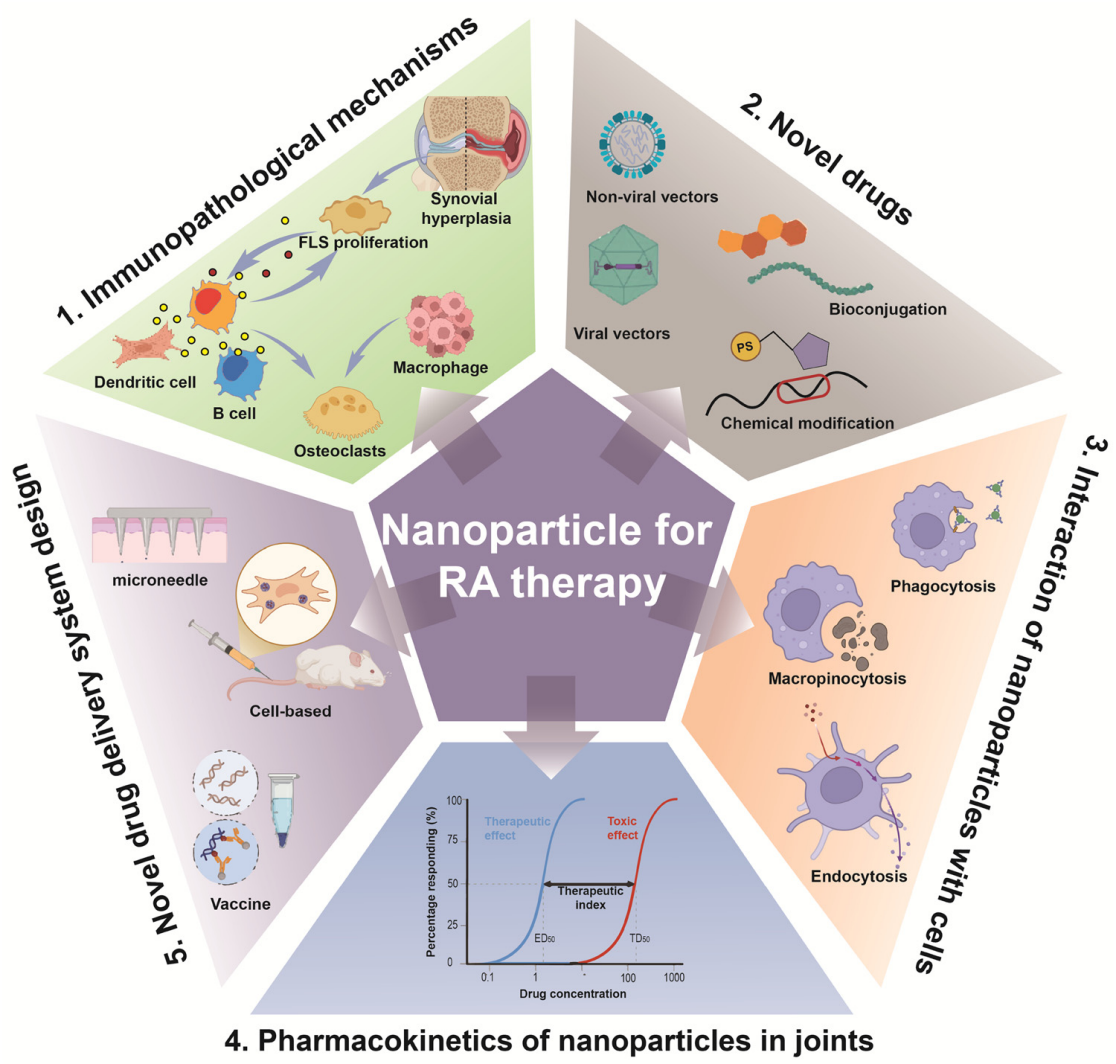
Representative research hotspots and frontiers in nanoparticle-based therapeutic approaches for RA.

The co-occurrence analysis of keywords, coupled with outbreak analysis, yielded valuable insights into the prevailing and emerging focus of joint dispersion in osteoarthritis research. As depicted in Figure 8, the keyword “inflammation” exhibited the highest frequency of citation bursts, underlining its foundational role in this domain of study. Figure 8c delineates prominent research clusters, encompassing key terms such as drug delivery systems, gold, and transdermal delivery. Furthermore, the comprehensive analysis reveals that nanoparticle research for RA therapy spans multiple research areas, indicative of its interdisciplinary nature. These findings serve as a gauge of current trajectories and frontiers in the field. The construction of the keyword co-occurrence network was predicated on the identification of keywords in the titles and abstracts of all amalgamated publications. Our findings are categorized into four principal sections: immunopathological mechanisms, novel drugs, interaction of nanoparticles with joint cells, pharmacokinetics of nanoparticles in joints, and novel nanoparticle-based drug delivery system design and evaluation. These results not only align with promising hotspots in the realm of nanoparticle research for RA therapy but also offer insights into prospective avenues for future research endeavors in this domain (Figure 10).

### 5.1 Immunopathological mechanisms

The co-occurrence analysis of keywords has identified four pivotal areas warranting further investigation: oxidative stress, dendritic cells, angiogenesis, and activated macrophages. These immunopathological mechanisms of RA serve as prime targets for nanoparticle systems, enabling precise drug delivery and system design. In a study conducted by Gravandi et al., an animal model of Freund’s complete adjuvant (FCA)-induced RA was constructed. The researchers demonstrated that FCA injection prolonged the immobility time of rats, as assessed by various tests including the open field test, acetone drop test, hot plate test, and Von Frey test. Additionally, they measured levels of glutathione (GSH), peroxidase, nitric oxide (NO), and activities of matrix metalloproteinase (MMP)-2 and MMP-9 [42]. In RA, there is a notable elevation in NO alongside a reduction in GSH and catalase levels. The experimental group in the study focused on rutin nanoparticles, which effectively regulated the oxidative stress milieu and cytokine levels. Furthermore, it inhibited MMP-9 activity while activating MMP-2, thus facilitating treatment. In another study, Prosperi et al. conducted a retrospective analysis to explore the advantages of nanoparticle systems in autoimmune diseases [43]. Nanoparticles represent a cutting-edge approach to drug delivery, leveraging precise target selectivity and enhanced drug-carrying capabilities within specific cells and tissues. This engineering finesse leads to optimized pharmacokinetics and heightened bioavailability of therapeutic agents, with a particular focus on innate immune cells such as macrophages, dendritic cells, and neutrophils. Given their inherent affinity for phagocytosis, nanoparticles hold promise as an immunotherapeutic platform for combating inflammatory and autoimmune diseases, potentially impeding processes like angiogenesis and macrophage activation.

### 5.2 Novel drugs

Leveraging the high efficiency of nanoparticle systems as carriers, we have engineered novel therapeutic drugs that exhibit superior efficacy when combined. Methotrexate, while a staple in the foundational treatment of RA, faces limitations due to the side effects and rapid drug efflux from the joint area. To address this challenge, Zykova et al. devised an emulsion comprising 50 nm phospholipid nanoparticles stabilized by glycyrrhizic acid [44]. The optimization of conditions for maximal methotrexate incorporation into phospholipid nanoparticles, controlled through HPLC, has significantly bolstered the therapeutic efficacy against RA compared to free methotrexate. Moreover, Diana et al. [45] showcased the anti-arthritic potential of NPs-CM by elucidating its role in inhibiting the NF-κB signaling pathway and curtailing the release of pro-inflammatory cytokines through comparative analysis. Additionally, the oral bioavailability of CM saw a substantial increase when formulated into Solutol-HS 15-stabilized nanoparticles, enabling the transition of CM-based RA therapy from intravenous to oral administration.

### 5.3 Interaction of nanoparticles with joint cells

The management of RA is directed toward pain relief, slowing disease progression, and enhancing joint mobility and function. While pharmacological strategies focus on symptom alleviation and improving quality of life, a definitive cure remains elusive. Intra-articular injections present several advantages for joint therapy, including heightened bioavailability and minimized systemic adverse effects [46]. Indeed, while this method enables swift clearance of the drug from the joint through capillary and lymphatic drainage, the dense extracellular matrix network in the cartilage may impede drug uptake and hinder the diffusion of drugs administered intra-articularly into target cells and sites. Consequently, this can impact the efficacy of drug utilization [47]. On the contrary, employing nanoparticles stabilizes the encapsulated drug, facilitating controlled drug release. This results in prolonged drug retention and mitigated site-specific toxicity, diffusion, and penetration into the extracellular matrix (ECM) and articular tissues. Additionally, nanoparticle-based delivery systems can actively or passively target specific sites, thereby amplifying therapeutic efficacy while diminishing the side effects associated with the loaded drug [48]. Within the joint, injected nanoparticles have the capacity to interact with cells such as immune cells, synoviocytes, and chondrocytes by either entering these cells or releasing loaded therapeutic agents. This intricate process is influenced by several factors, including nanoparticle size, shape, charge, surface functionalization, and composition, as well as the biological and pathological microenvironment within the joints [49].

Protein corona formation is a phenomenon wherein proteins from the biological environment competitively bind to the surface of nanoparticles, forming a “corona” layer [50,51]. This can alter the nanoparticles’ physicochemical properties, including size, charge, and surface characteristics, and subsequently affect their interactions with biological tissues and cells [52]. In the context of RA, this issue is particularly relevant as the protein corona can influence the behavior of nanoparticles within inflamed joint tissues. Specifically, disorders like autoimmune hemolytic anemia, commonly associated with RA, may alter the binding affinity of proteins to nanoparticle surfaces, resulting in changes in nanoparticle stability, cellular uptake, and drug delivery efficiency [53]. Furthermore, such modifications could impact the immunological response, potentially affecting the therapeutic outcomes of nanoparticle-based drug delivery systems [54]. Understanding the dynamics of protein corona formation is crucial for optimizing the design and effectiveness of nanoparticles used in RA therapy, ensuring their targeting accuracy, and minimizing off-target effects [55,56].

### 5.4 Pharmacokinetics of nanoparticles in joints

Lymphatic drainage facilitates the rapid clearance of drugs from the joint, a process largely influenced by the size of the molecule. Nanoparticles, being larger in size compared to free drug molecules, tend to be retained in the joint for a longer period of time, thereby potentially extending their therapeutic effects [57].

Furthermore, the physical and chemical properties of nanoparticles, including size, shape, and surface properties, play a crucial role in determining their residence time within the joint. Research by Rothenfluh et al. has shown that nanoparticles with tissue-specific targeting abilities exhibit enhanced residence time in the joint space [58]. Indeed, extending the joint retention of nanoparticles is achievable through biomolecule targeting, such as collagen type II. For instance, by incorporating a collagen II-binding peptide into the nanoparticles, their retention in the joint can be prolonged significantly. Studies have shown that nanoparticles modified with a collagen II-binding peptide can remain in the joint for up to 7 days, whereas those with a scrambled peptide are typically cleared within 6–8 h [59]. The principal degradation pathways of nanoparticles encompass chelation, hydrolysis, redox reactions, and enzymolysis [48]. Lymphatic and capillary drainage through the synovium beneath the periarticular joint aids in the elimination of nanoparticles and their encapsulated drugs from the joint cavity. While small particles tend to exit the joint via capillaries, the lymphatic pathway clears nanoparticles and their degradation products regardless of their size. In patients with RA, enhanced synovial lymphatic blood flow due to systemic inflammation and autoimmune dysfunction leads to increased clearance of large particles or molecules, although further confirmation of the nanoparticle removal mechanism is warranted. Given the limited skin permeability of methotrexate (MTX), Amarji et al. [60] devised the microemulsions nanoparticle system to target the drug to specific areas within the stratum corneum, epidermis, and dermis of the skin, thereby reducing systemic absorption. This underscores its potential as a novel vehicle for the topical delivery of MTX in therapeutic and safety applications.

### 5.5 Novel nanoparticle-based drug delivery system design and evaluation

The advancement of biocompatible nanoparticles for *in vivo* molecular imaging and targeted therapies has garnered significant interest across various scientific, engineering, and biomedical disciplines, including the realm of RA therapy. Recent studies highlight that pegylated gold nanoparticles, which involve colloidal gold coated with a protective layer of polyethylene glycol (PEG), demonstrate exceptional *in vivo* biodistribution and pharmacokinetic properties following systemic injection [61,62]. In contrast to cadmium-containing quantum dots and other nanoparticles that may pose toxicity or elicit immune responses, gold colloids exhibit minimal to no long-term toxicity or adverse effects *in vivo* [63]. Since certain genes within a drug delivery system have the potential to inhibit undesired gene expression in specific target cells, Stephen et al. encapsulated dexamethasone into PLGA nanoparticles [63]. The drug-loaded PLGA nanoparticles were complexed with polyethyleneimine/siRNA to suppress the expression of undesirable genes and proteins linked to arthritis. The transfection of dexamethasone-loaded and COX-2 siRNA-complexed PLGA nanoparticles was investigated to assess their impact on the expression of arthritis-related genes and the reduction in protein expression. Effective treatment of RA faces challenges due to the scarcity of drugs that specifically target inflamed joints. Liposomes, nanoparticles, and conventional micelles containing limited drug amounts may exhibit instability in circulation, resulting in uncontrolled drug release kinetics. Li et al. introduced a novel drug delivery system comprising pH-sensitive polymeric micelles built on an acid-labile hydrazone bond. Amphiphilic conjugates of a PEG-based derivative and the hydrophobic drug prednisolone (PD) self-assembled into PD micelles with a drug loading of 19.29%. Upon encountering the acidic environment of the synovial fluid, hydrolysis ensued, liberating free PD. The enhancement of joint concentration, based on the area under the concentration-time curve, was 4.63-fold, underscoring the potential of PD micelles for targeted drug delivery in inflammatory diseases [64].

### 5.6 Impact of work instability (WI) on RA patients

As highlighted in recent studies, WI can have significant consequences for individuals with RA [65]. WI refers to the risk of continuing employment being threatened due to a mismatch between an individual’s functional abilities and the demands of their job. This is especially relevant for RA patients, who often experience fluctuating physical abilities that may affect their ability to meet the demands of their job roles. A study developed the Work Instability Scale (WIS), which provides a tool for assessing the levels of WI in individuals with RA [66]. This scale classifies WI into three bands – low, medium, and high risk – offering a practical method for identifying those at risk of work-related disability.

In RA patients, the consequences of WI are multifaceted [67]. On the one hand, it can exacerbate the physical and psychological burden of the disease, leading to increased stress, decreased morale, and reduced productivity. On the other hand, addressing WI through targeted management strategies can play a crucial role in improving the patient’s overall well-being, job retention, and treatment outcomes. This aligns with findings in organizational behavior research, which emphasizes the importance of authentic leadership and the role of job fit in fostering positive employee outcomes. Authentic leadership has been shown to significantly enhance employee organizational identification and felt obligation, creating a supportive work environment that can buffer against the adverse effects of WI [68].

Leaders who foster an environment of understanding, support, and job flexibility can significantly enhance the mental and physical health of employees with RA. Proper job fit, as discussed in both the RA WI study and leadership literature, emerges as a crucial factor that can help employees navigate the challenges of WI while minimizing the risk of work-related disability [68]. Incorporating strategies for identifying and addressing WI, such as using the WIS for RA patients, along with promoting authentic leadership and ensuring appropriate job fit, could substantially improve the overall work experience for RA patients, as well as their job retention and health outcomes [69].

### 5.7 Role of nanomaterials in degradation of chemical drugs for autoimmune diseases

Nanomaterials have shown promise in RA therapy and in the degradation of various pharmaceutical agents used to treat autoimmune diseases, including RA [70]. They have demonstrated effectiveness in the photocatalytic degradation of analgesics, mucolytics, and anti-inflammatory drugs commonly prescribed for managing autoimmune conditions like RA. One study highlights how nanomaterials can efficiently degrade drugs used in treating diseases like SARS-CoV-2 and autoimmune disorders, preventing environmental contamination [71].

Pharmaceuticals such as painkillers, antibiotics, and anti-inflammatory drugs persist in the environment, contaminating both terrestrial and aquatic ecosystems, posing risks to wildlife. Nanomaterials can address this issue by enabling the safe and efficient degradation of these drugs, thus contributing to environmental conservation [72–74]. This research underscores the dual benefits of nanomaterials: their potential in RA treatment and their role in sustainable pharmaceutical waste management. By degrading drugs that enter ecosystems through wastewater, nanomaterials offer a sustainable solution to mitigating pharmaceutical contamination. These findings highlight the broader significance of nanomaterials in both healthcare and environmental sustainability.

### 5.8 Sjögren’s syndrome (SS) and its impact on oral health in RA patients

SS is an autoimmune disorder frequently associated with RA, in which chronic inflammation damages exocrine glands, primarily the salivary and lacrimal glands, leading to reduced secretions and severe oral and ocular dryness [75,76]. The resultant xerostomia (dry mouth) not only impairs the ability to chew, swallow, and speak but also significantly increases the risk of dental caries, periodontal disease, and oral infections [77]. Saliva plays a crucial role in maintaining oral homeostasis by neutralizing acids, washing away food debris, and providing antimicrobial peptides; therefore, its depletion predisposes individuals to a heightened risk of oral pathology [78]. Furthermore, recent studies highlight that SS in RA patients is often underdiagnosed due to its overlapping symptoms with other autoimmune conditions. Advanced imaging techniques, such as Cone Beam Computed Tomography (CBCT), have demonstrated superior sensitivity in detecting glandular abnormalities compared to traditional physical examinations [79]. In particular, CBCT enables precise assessment of salivary gland calcifications, while power Doppler ultrasound can visualize real-time inflammation in the affected glands, making these tools invaluable for early detection and targeted treatment [80,81].

Beyond oral health deterioration, SS-related salivary gland dysfunction may contribute to dysphagia, which can exacerbate nutritional deficiencies and further compromise patient well-being [82]. Current therapeutic approaches focus on symptom management, including artificial saliva substitutes, systemic secretagogues (e.g., pilocarpine), and localized treatments like low-level laser therapy to enhance salivary gland function [83,84]. The effectiveness of alcohol-free mouthwashes (e.g., Biotene) and fluoride rinses in alleviating xerostomia symptoms is well-documented, along with sugar-free lozenges or gum to stimulate residual salivation [85,86]. Given the increasing recognition of the role of SS in RA, more comprehensive patient education and interdisciplinary collaboration between rheumatologists, dentists, and oral medicine specialists are crucial. Regular dental visits, rigorous oral hygiene routines, and early imaging-based diagnostics should be incorporated into routine RA management to mitigate the long-term consequences of SS-induced oral complications [87,88].

### 5.9 Impact of cancer and occupational risks on RA patients

RA patients are at higher risk of occupational cancers due to prolonged exposure to environmental and occupational hazards [89]. The WHO estimates that over 200,000 deaths annually are linked to workplace cancer exposures, including air pollution, toxic chemicals, pesticides, heavy metals, and radiation. Given RA’s potential to weaken the immune system, patients may be more susceptible to these carcinogens. Workers in industries with heavy metals or chemicals face an increased cancer risk due to carcinogenic exposures [90,91]. Given cancer’s multifactorial nature, including genetic, environmental, and lifestyle factors, early detection through regular screenings is vital for reducing cancer risks in RA patients. Preventive measures should include occupational safety audits, proper personal protective equipment use, and minimizing harmful exposures. Public health strategies to reduce environmental pollution can lower cancer risks for both RA patients and the general population [92]. Therefore, a comprehensive healthcare approach should address the combined impact of rheumatic diseases and environmental/occupational exposures, with public health initiatives focusing on cancer risk reduction.

## 6 Conclusion

In conclusion, this study provides a comprehensive bibliometric and visual analysis of nanoparticle-based therapeutic approaches for RA from 2003 to 2023. By analyzing quantitative outputs, collaborative networks, and key research themes, we identified global trends and emerging hotspots, such as drug delivery systems, joint cell interactions, and nanoparticle pharmacokinetics. Notably, China has become a dominant player in publications, while the United States stands out for its high impact in terms of H index and citations. The findings underscore the significant progress in understanding immunopathological mechanisms and the development of novel nanoparticle-based therapies. This study highlights the growing importance of nanoparticles in RA treatment, offering a foundation for future research and innovation in the field. The insights presented here not only aid researchers in navigating the evolving landscape but also emphasize the potential of nanoparticle systems to transform RA therapy.
